# The tRNA-Derived Fragment-3017A Promotes Metastasis by Inhibiting NELL2 in Human Gastric Cancer

**DOI:** 10.3389/fonc.2020.570916

**Published:** 2021-02-16

**Authors:** Linhao Tong, Weixu Zhang, Bicheng Qu, Fei Zhang, Zhonghua Wu, Jinxin Shi, Xiaowan Chen, Yongxi Song, Zhenning Wang

**Affiliations:** Department of Surgical Oncology and General Surgery, Key Laboratory of Precision Diagnosis and Treatment of Gastrointestinal Tumors, Ministry of Education, The First Affiliated Hospital of China Medical University, Shenyang, China

**Keywords:** tRNA-derived fragment, gastric cancer, lymph node metastasis, NELL2, molecular mechanism

## Abstract

tRNA-derived fragments (tRFs) are a new classification of small non-coding RNAs (sncRNAs) derived from the specific cleavage of precursors and mature tRNAs. Accumulating recent evidence has shown that tRFs are frequently abnormal in several cancers. Nevertheless, the role of tRFs in gastric cancer and its mechanism remain unclear. In this study, we found abnormal expression of tRF-3017A (derived from tRNA-Val-TAC) in gastric cancer tissues and cell lines and confirmed its effect on promoting the invasion and migration of gastric cancer cells through functional experiments *in vitro*. Analysis of clinicopathologic data showed patients with higher tRF-3017A were associated with significantly higher lymph node metastasis. Mechanistic investigation implies that tRF-3017A regulates the tumor suppressor gene NELL2 through forming the RNA-induced silencing complex (RISC) with Argonaute (AGO) proteins. In this study, we found that higher tRF-3017A were associated with significantly higher lymph node metastasis in gastric cancer patients and the tRF-3017A may play a role in promoting the migration and invasion of gastric cancer cells by silencing tumor suppressor NELL2.

## Introduction 

Gastric cancer (GC) is one of the most common human cancers, which is the second leading cause of cancer death worldwide, and its global burden is increasing (1, 2). In recent years, the incidence and mortality rates of GC have been declining (3). However, GC still has the worst outcome of all solid organ tumors, due to the frequent occurrence of late lymph node metastasis or distant metastasis (4). Because the treatment of advanced GC is limited and the prognosis is poor, it is urgent to find new biomarkers and prognostic indicators to reflect the disease status and develop more therapeutic targets for this deadly disease.

tRNA-derived fragments (tRFs) are ubiquitous in all areas of biology. As short non-coding RNAs, they are abundant and heterogeneous (5–7). Increasing studies have shown that tRFs aren’t products from random degradation. Instead they are producted by specific cleavage of multiple pre-tRNAs and mature tRNAs by different ribonucleases (8–11). tRFs are classified into two main subgroups: shorter tRFs of length 14–36 nt and longer tRFs of lengths 30–40 nt (12–14). Angiogenin (ANG) and other RNase cleave specific tRNAs and create longer tRFs (15). Dicer and ANG cleave specific tRNAs and produce shorter tRFs (16). Although the naming of tRFs has not yet been unified, it is certain that there are differences in the biological sources and functions of tRFs in different subgroups (17). It has been indicated in studies that tRFs play important roles in oncogenesis and cancer progression (16, 18–21). tRFs can regulate tumor progression by competitive binding of RNA binding proteins (22–24). It has also been reported that tRFs can play an RNA silencing role similar to that of miRNA, which is, mRNA silencing by forming RISC with AGO protein (25, 26). Another study reported that a specific tRNA-derived small RNA (tsRNA) named LeuCAG 3’ tsRNA binds to mRNAs of ribosomal proteins to enhance efficient translation (27). In summary, the main biological functions of tRFs include regulation of gene expression, protein translation and various cellular activities (28). Morever, recent studies have suggested that tRFs can potentialy serve for prediction in breast cancer (29–32), clear cell carcinoma (33, 34), colorectal cancer (35, 36), and prostate cancer (37–39). Therefore, much attention has been paid to these tRNA derivatives for cancer predictor and therapeutic targets (20).

Abnormal expression of tRNA-derived fragments in GC was detected through tRF&tiRNA array and we chose this upregulated tRF-3017A for further study. tRF-3017A, a kind of specific degradation product of mature tRNA-Val-TAC 3’ end, is composed of 19 nt (5’-AGCCCCAGTGGAACCACCA’). Based on qRT-PCR and clinicopathologic data analysis, we suggested that the expression level of tRF-3017A was abnormally increased in patients with lymph node metastasis of GC. The results of functional experiments showed that tRF-3017A can promote GC cells by targeting and regulating NELL2, its downstream mRNA. Nerve epidermal growth factor-like like protein (NELL) was originally discovered in chickens as a polymeric and multimodular extracellular glycoprotein (40). Two NELL mammalian homologues, named NELL1 and NELL2, have been found in the human fetal brain cDNA library (41). NELL2 was found enriched in the nervous system in the beginning and was known to be involved in neural development (41–44). Previous studies have shown that NELL2 is enriched in normal nerve cells compared with nervous system tumors (45) and that it inhibits cancer cell migration in renal cell carcinoma (46).

In this present study, we detected tRF-3017A expression level in tissues of GC patients and GC cell lines, and further analyzed the relevance between tRF-3017A and clinicopathologic features of GC. Biological functions of tRF-3017A were explored by functional experiments which identified its effect on migration and invasion. Furthermore, we identified a potential molecular regulatory relationship between tRF-3017A and NELL2 in GC progression.

## Materials and Methods

### Tissues

Eighty-seven GC tissues and matched-paired noncancerous adjacent tissues (NATs) were obtained from GC patients who underwent surgical treatment in the First Affiliated Hospital of China Medical University (Shenyang, China) between 2016 and 2017. All of these GC patients were confirmed by pathological diagnosis and agreed after being informed according to ethical guidelines. No patients were treated with chemoradiotherapy or targeted therapy before the operation. Matched-paired NATs were obtained from areas more than 5 cm away from the lesion. All tissue samples were put into liquid nitrogen immediately after separation and then transferred to −80°C for long-term storage. The research ethics committee of the First Affiliated Hospital of China Medical University permitted this study which conformed to the criteria of the declaration of Helsinki.

### Cell Culture

The human gastric mucosal epithelial cell line (GES-1) was obtained from BeNa Culture Collection (Henan, China). The human GC cell lines MGC-803 and HGC-27 were purchased from Shanghai Institutes for Biological Sciences, China Academy of Science (Shanghai, China). AGS, MKN-45, and SNU-16 cells were obtained from the American Type Culture Collection (Manassas, Virginia). All cells were incubated in RPMI 1640 medium (HyClone) supplemented with 10% fetal bovine serum (FBS) and cultured at 37°C in an atmosphere containing 5% CO_2_ (Thermo, Waltham, MA, USA).

### Microarray Analysis

Tissues and matched NATs of 10 patients with GC were prepared for the nrStarTM Human tRF&tiRNA PCR Array (Arraystar, Lnc. Rockville, MD 20850 USA. Cat#: AS-NR-002) analysis. Microarray hybridization and samples preparation followed manufacturer’s standard protocols.

### RNA Extraction and Quantitative Real‐Time PCR (qRT-PCR)

According to the manufacturer’s agreement, the total RNA of GC tissues and cells was extracted by TRIzol reagent (Invitrogen, USA). Mir-X™ miRNA First-Strand Synthesis Kit (Clontech) or PrimeScript RT reagent Kit (Takara) was used to synthesize the complementary DNA (cDNA). TB Green^®^ (RR820A, Takara) was used to identify expression level of tRF-3017A and mRNAs on the Light Cycler 480 II Real-Time PCR system (Roche Diagnostics). The program consists of a 95°C temperature cycle for 5 s, 60°C for 20 s, and 55°C for 30 s, repeating 45 times. Samples were analyzed in triplicate, and melting curve analysis were used to identify the specificity of the primers. Cycle threshold (Ct) was based on total cycles required for the TB Green^®^ signal to cross the threshold. The relative expression of tRF-3017A in all samples were calculated using the Ct method normalized to RNU6B (U6) and Glyceraldehyde phosphate dehydrogenase (GAPDH) for mRNAs. Samples with a Ct >38 were considered negative. The primers were customized using Sangon Biotech, and sequences are shown in **Table S1**. After the reaction, 2^-ΔΔCt^ method was used to analyze the data results, and the formula was shown as follows: ΔΔCt = ΔCt_tumor[Ct(target)-Ct(reference)]_-ΔCt_NATs[Ct(target)-Ct(reference)]_.

### Cell Transfection

According to the cellular tolerance, six-well plates with 2 ml culture medium were used to plate 2×10^5^ cells per well. Cells were transiently transfected by 50 nM tRF-3017A mimics or corresponding negative control-mimics (NC-mimics) and 100 nM tRF-3017A inhibitor or NC-inhibitor (GenePharma) by utilization of Lipofectamine 3000 Reagent (Thermo Fisher Scientific). Short interfering RNAs (siRNAs) targeting NELL2 and pEX3 plasmid for overexpressing NELL2 were synthesized by RiboBio and GenePharma. The final transfection concentration of pEX3-NELL2 and si-NELL2 was 50 and 100 nM, respectively. Sequences of transfection reagents are shown in **Table S1**. After 48 h transfection, cells were harvested and used in subsequent experiments.

### Transwell Assay

Transwells (REF3422, Corning, NY, USA) were performed to transwell migration assay. 5×10^4^ cells transfected with tRF-3017A mimics or tRF-3017A inhibitor after 48 h were cultured with 200 μl RPMI medium (without FBS) in the upper chamber, and 600 μl culture medium with 10% FBS was added to the bottom chamber. Cotton swabs were cautiously used to remove the non-migratory cells from the upper chamber after 24 h of incubation at 37°C with 5% CO_2_. After staining with hematoxylin and eosin (H&E), the remaining cells were captured in 9 randomly selected visual fields by utilization of a Leica DM3000 microscope (Leica, Wetzlar, Germany). Color grab comparison method of Software Image-Pro 6.0 was used to count the remaining cells. For transwell invasion experiments, Matrigel (356234, MA, USA) was added to the upper chamber based on the above scheme.

### Wound Healing Assay

2.5×10^4^ cells transfected with tRF-3017A mimics or tRF-3017A inhibitor after 48 h were plated in 12-well plates and cultured all-night. The single-cell layer was nicked using the tip of a 200-microlitre pipettor. Before adding the new medium (without FBS), PBS was used to remove floating cells and scraped cell fragments. Areas of wound were displayed at 200x magnification under an inverted light microscope (Leica DMI3000B). Images were captured at 0 and 24 h after the scratch was made. The scratch area coverage, which reflects the migration ability of GC cells, was analyzed by Image-J software (Media Cybernetics, Rockville, MD, USA).

### Cell Counting Kit-8 Proliferation Assay

The Cell Counting Kit-8 (CCK-8) (SA618, Dojindo Laboratories, Kumamoto, Japan) was used to measure the capacity for cellular proliferation according to the manufacturer’s instructions. 2×10^3^ cells of HGC-27 were seeded into 96-well culture plates for 24, 48, 72, and 96 h, respectively. The microplate reader (Bio-Rad, Hercules, CA, USA) was used to determine the optical density at a wavelength of 450 nm.

### Western Blot

Total protein was extracted *via* Total Protein Extraction kit (T9300A, BCA Protein Assay Kit, Takara). SDS-PAGE gels (KGMG010W10, KeyGen) and polyvinyl difluoride (PVDF) membranes (ISEQ00010, Millipore, MA, USA) are used for protein electrophoresis and transfer, respectively. 5% free fat milk emulsion was used to block the membrane at room temperature for 2 h. Then the primary antibody was incubated overnight at 4°C. Primary antibodies are NELL2 antibody 1:1,000 dilution (ab181376, rabbit monoclonal antibody, Abcam, CA, USA) and GAPDH antibody 1:5,000 dilution (ab21612, rabbit polyclonal antibody, Abcam, CA, USA). Secondary antibodies which anti-rabbit (1:5,000, ZB2301, ZSGB-BIO, Beijing, China) were added at 25°C for 1.5 h, then membranes were imaged by GelCapture software (DNR Bio-Imaging Systems). For experiments based on GC tissue samples, MinuteTM (Tissues Total Protein Extraction Kit for WB, Cat No. SD-001, MN, USA) was used to extract total protein.

### RNA Immunoprecipitation (RIP) Assay

The Magna RIP RNA-binding protein immunoprecipitation kit (17-700, Millipore, MA, USA) was used to perform the RIP assay for Ago2. Anti-Ago2 antibody (ab32381, Abcam, CA, USA) and control IgG were used for the RIP assay. The expression of tRF-3017A and NELL2 immunoprecipitated by Ago2 were evaluated by qRT-PCR.

### Dual-Luciferase Reporter Assay

To determine the target regulating relationship between tRF-3017A and NELL2 mRNA, a luciferase reporter assay was implemented using AGS cells. 3 ‘UTR of NELL2 mRNA including the tRF-3017A binding site and it’s mutant construct were inserted in the luciferase reporter vector of pmirGLO. The luciferase reporter was cotransfected with NELL2-3’UTR fusion vector and tRF-3017A mimics, inhibitor and corresponding NC. Cells were harvested 48 h later. And then the luciferase reporter assay (E1910, Promega, USA) and Infinite M200 PRO microplate reader (Tecan) were used to detect the luciferase activities (firefly and renilla).

### Statistical Analysis

All data were analyzed by SPSS version 21.0 (Chicago, IL, USA), GraphPad Prism, Image-Pro Plus, and Image J software. The histogram drawn according to -ΔΔCt is used to describe the expression level of tRF-3017A in 87 GC patients. The scatter plot drawn according to -ΔCt and paired t test was used to describe the relative expression of tRF-3017A in 87 patients with cancer tissue and its paired NATs. Chi-square test and ROC analysis were used to analyze the correlation between expression of tRF-3017A and clinicopathologic data of 87 GC patients. The relative expression of tRF-3017A and clinicopathologic data in 87 patients were used for ROC analysis. Pearson correlation was performed to determine correlation coefficients. All experiments were accomplished with the lowest three times. Mean value ± standard deviation (SD) was used to list Data. The differentiation between two groups was compared with the Student’s t-test.

## Results

### Expression of tRF-3017A Is Increased in GC Tissues and Cell Lines

**Figure 1:**
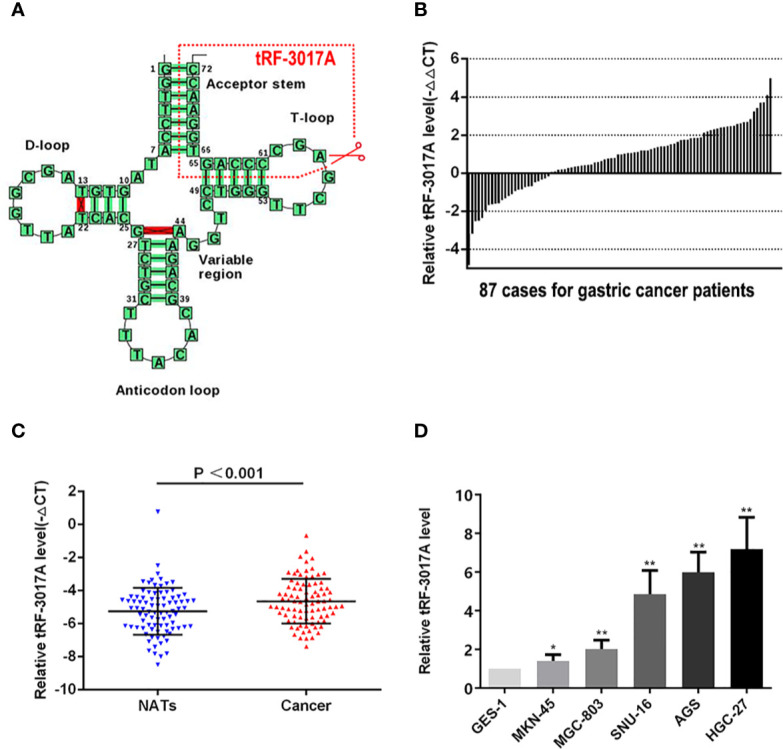
Expression of tRF-3017A in GC tissues and cell lines. **(A)** tRF-3017A is a 3’ fragment of mature tRNA-Val-TAC. The specific cleavage site is located at the T-loop. Image from tRNADB (http://trna.bioinf.uni-leipzig.de/DataOutput/Result?ID=tdbD00011005). **(B)** Expression level (-ΔΔCt) of tRF-3017A was quantified in GC tissues compared with matched-paired NATs among 87 GC patients by qRT-PCR, and U6 RNA was used as a qRT-PCR control for tRF-3017A. Each column represents up/down-regulated tRF-3017A expression in one GC patient. **(C)** Expression of tRF-3017A was upregulated (-ΔCt, paired t test) in GC tissues relative to matched-paired NATs among 87 GC patients by qRT-PCR. **(D)** Expression of tRF-3017A in GC cell lines. GES-1, human gastric mucosal epithelial cell, was used as a qRT-PCR control. *P < 0.05; **P < 0.01.

According to the tRF&tiRNA PCR array data, we analyzed the differential expression of tRNA fragments (fold change >2 and P <0.05) and found that six were upregulated and 12 were downregulated in GC tissues compared with NATs. Statistically significant gene results were shown in **Figure S1A** and **Table S3**. An upregulated tRF, tRF-3017A was selected for following study. tRF-3017A is a degradation product of mature tRNA-Val-TAC which specific cleave at the 3’ end of T-loop (**Figure 1A**). We investigated the expression of tRF-3017A using qRT-PCR in 87 cases of GC tissues and matched-paired NATs and found upregulated tRF-3017A in 62 of 87 (71.2%) pairs of GC tissue relative to matched-paired NATs (-ΔΔCt, **Figure 1B**). Based on paired t test, the expression of tRF-3017A was higher in GC tissues than in matched-paired NATs (-ΔCt, P < 0.001, **Figure 1C**). We then detected the expression level of tRF-3017A in GC cell lines (MKN-45, MGC-803, SNU-16, AGS, and HGC-27), using its expression level in GES-1 as a reference. We found increased expression of tRF-3017A in GC cells (**Figure 1D**).

Chi-square test was used to analyze the clinicopathologic data of 87 GC patients to further explore the potential clinical value of tRF-3017A expression level and clinicopathologic features (**Table 1**). We found that higher tRF-3017A expression level was significantly associated with higher lymph node metastasis (P = 0.016). ROC curves were carried out to identify if tRF-3017A could function as a diagnostic tool to differentiate GC tissues from matched-paired NATs or to indicate more detailed clinicopathologic features. Unfortunately, the tRF has not demonstrated satisfactory diagnostic efficacy (**Figures S1B, C**).

**Table 1:** Correlation between tRF-3017A expression and clinicopathological factors in tissue samples of GC patients (n = 87).

Variable	tRF-3017A expression		P-value
	Low no. (%)	High no. (%)	
Age			0.144
<60	11 (35.5)	29 (51.8)	
≥60	20 (64.5)	27 (48.2)	
Gender			0.126
Male	21 (37.7)	46 (82.1)	
Female	10 (32.3)	10 (17.9)	
Tumor size (cm)			0.411
<4.5	15 (48.4)	22 (39.3)	
≥4.5	16 (51.6)	34 (60.7)	
Histologic grade			0.807
Well/moderately	13 (41.9)	25 (44.6)	
Poorly	18 (58.1)	31 (55.4)	
pT stage			0.558
pT1	2 (6.5)	5 (8.9)	
pT2	5 (16.1)	5 (8.9)	
pT3	9 (29)	12 (21.4)	
pT4	15 (48.4)	34 (60.7)	
pN stage			0.016*
pN-	15 (48.4)	13 (23.2)	
pN+	16 (51.6)	43 (76.8)	
pTNM stage			0.064
I	4 (13)	8 (14.3)	
II	13 (41.9)	9 (16.1)	
III	13 (41.9)	36 (64.3)	
IV	1 (3.2)	3 (5.4)	
Invasion into lymphatic vessels			0.066
Absent	11 (35.5)	10 (17.9)	
Present	20 (64.5)	46 (82.1)	
Venous invasion			0.691
Absent	27 (87.1)	47 (83.9)	
Present	4 (12.9)	9 (16.1)	

tRF-3017A, tRNA-derived fragment 3017A; pT, pathological Tumor; pN, pathological Node; pTNM, pathological Tumor-Node-Metastasis; *P < 0.05. Bold value indicates P‐value was lower than 0.05 with statistical significance.

Collectively, these results suggest that up-regulated expression of tRF-3017A may play a role in the metastasis of GC.

### tRF-3017A Promotes the Invasion and Migration of GC Cells

**Figure 2:**
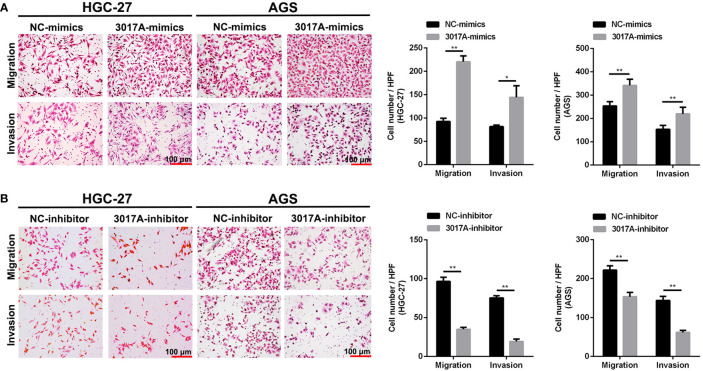
Transwell assays verified the effect of tRF-3017A on migration and invasion of GC cells. **(A)** Migration and invasion of GC cell lines HGC-27 and AGS were investigated after tRF-3017A-mimics or NC-mimics. **(B)** Migration and invasion of GC cell lines HGC-27 and AGS were investigated after tRF-3017A-inhibitor or NC-inhibitor. Representative images and bar graphs were depicted. Data are shown as mean ± SD. NC, negative control. *P < 0.05; **P < 0.01.

**Figure 3:**
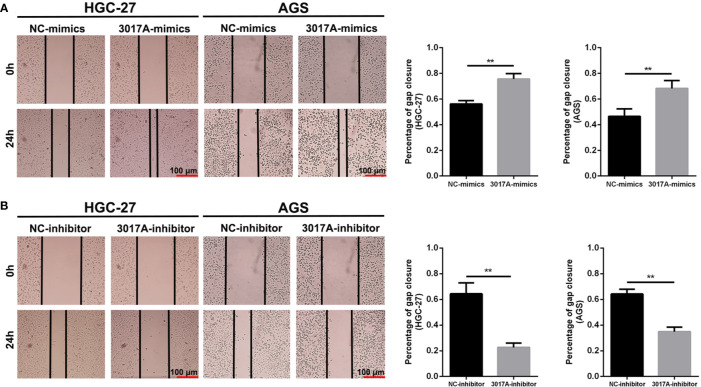
The scratch wound healing assay on GC cell lines. **(A)** The scratch wound healing assay was used to determine the cell motility ability in GC cell lines HGC-27 and AGS transfected with tRF-3017A-mimics or NC-mimics. **(B)** The scratch wound healing assay was used to determine the cell migration ability in GC cell lines HGC-27 and AGS transfected with tRF-3017A-inhibitor or NC-inhibitor. Representative images and bar graphs were depicted. Data are shown as mean ± SD. NC, negative control. **P < 0.01.

To further explore whether tRF-3017A could regulate biological function in GC cells, we knocked down and overexpressed tRF-3017A in HGC-27 and AGS cells, and qRT-PCR was used to detect the overexpression efficiency (**Figure S1D**). Transwell assay was performed to evaluate the migratory and invasive capacities of GC cells transfected tRF-3017A mimics or inhibitor and their matching NC. As shown in **Figure 2A**, GC cells treated with 3017A-mimics showed significantly enhanced invasion and migration abilities. In contrast, GC cells treated with 3017A-inhibitor had markedly weaker abilities than in the NC group (**Figure 2B**). A wound healing assay was then conducted to observe whether tRF-3017A could affect the motility of GC cells. Different scratch healing rates in wound healing analysis showed that motile ability was significantly enhanced after tRF-3017A overexpression and decreased after tRF-3017A knockdown (**Figures 3A, B**). CCK-8 proliferative assays was performed to detected how tRF-3017A affects GC cell proliferation. However, proliferation of GC cells were not affected by knockdown/overexpression of tRF-3017A (**Figure S1E**).

### tRF-3017A May Play a Role in Regulating the Migration and Invasion of GC Cells by Targeting NELL2

**Figure 4:**
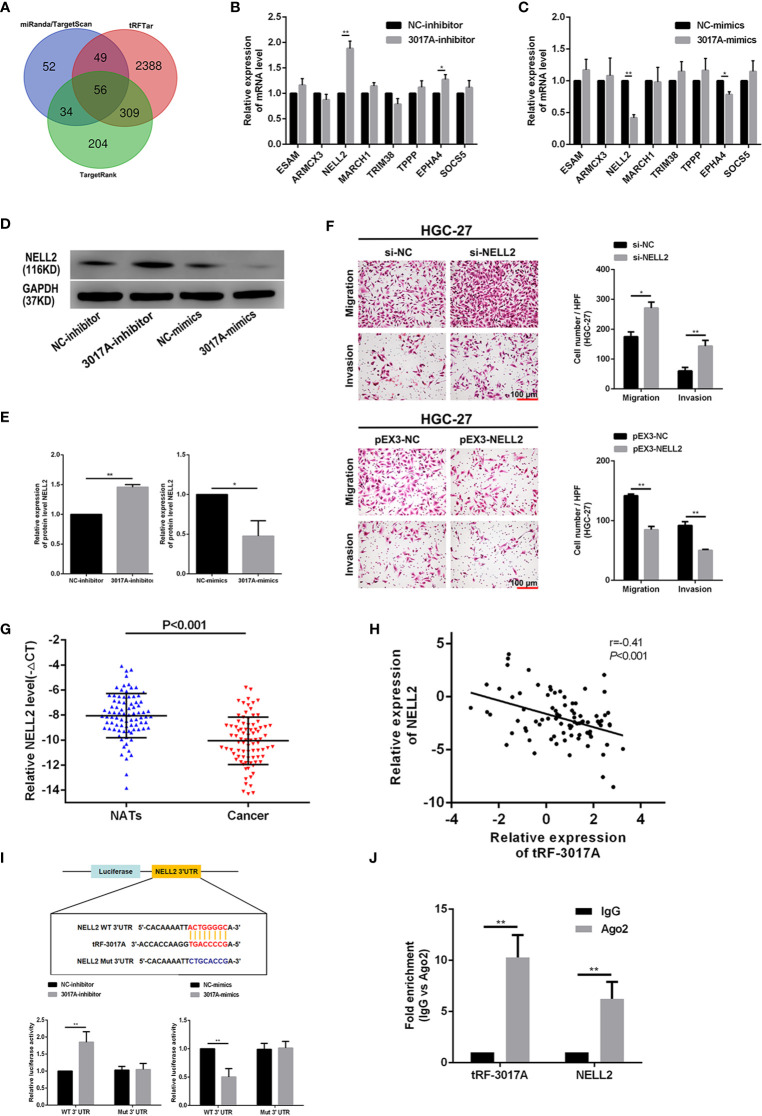
tRF-3017A may play a role in regulating the migration and invasion of GC cells by regulating NELL2. **(A)** Venn diagram evaluated the overlapped genes among miRanda, TargetScan, TargetRank, and tRFTar predictions. **(B, C)** The expression levels of eight predicted target genes were performed to detect in AGS cell line after transfection with tRF-3017A-inhibitor or mimics by qRT-PCR. **(D, E)** The expression levels of NELL2 were detected in AGS cell line after transfection with tRF-3017A-inhibitor or mimics by western blot. **(F)** GC cell migration and invasion ability were detected after transfected with si-NELL2 and pEX3-NELL2. **(G)** Expression of NELL2 was downregulated (-ΔCt, paired t test) in GC tissues relative to matched-paired NATs among 84 GC patients by qRT-PCR. **(H)** Correlation analysis of relative expressions of tRF-3017A and NELL2 and “r” is the correlation coefficient. **(I)** The luciferase activity of wild type NELL2 3’UTR or mutant NELL2 3’UTR after transfection with tRF-3017A mimics or 3017A-inhibitor and their corresponding NC in AGS cell line. **(J)** The result of the RIP based on Ago2 showed that tRF-3017A may exert its miRNA-like silencing effect by combining Ago2, thus targeting NELL2. Representative images and bar graphs were depicted. Data are shown as mean ± SD. NC, negative control. *P < 0.05; **P < 0.01.

We used the 3’ UTR complementary binding principle and mRNA target prediction algorithms to predict downstream target genes of tRF-3017A using online databases, and to investigate the molecular mechanism of tRF-3017A in regulating GC cells. As shown in **Figure 4A**, we evaluated target gene results that overlapped between miRanda, TargetScan, TargetRank, and tRFTar and reviewed available literature to improve reliability and narrow the prediction range. We comprehensively considered structure scores, free energy ranking and reports of existing studies, and selected eight higher ranking genes, ESAM, ARMCX3, NELL2, MARCH1, TRIM38, TPPP, EPHA4, and SOCS5. Preliminary validating results by qRT-PCR indicated that NELL2 changed the most of all and that its expression levels were upregulated after transfection with tRF-3017A inhibitor or downregulated after transfection with tRF-3017A mimics (**Figures 4B, C**). Although there was also a statistically change in EPHA4, NELL2 was selected for further study due to its most significant change. To verify the regulatory relationship between tRF-3017A and NELL2 at the protein level, we transfected GC cells with tRF-3017A mimics or inhibitors and detected NELL2 expression level by Western blot. tRF-3017A overexpression significantly decreased protein expression of NELL2 in GC cells. In contrast, tRF-3017A inhibition significantly increased protein expression of NELL2 in GC cells (**Figures 4D, E**). Transwell assay was carried out to validate the biological function of NELL2 in HGC-27. As shown in **Figure 4F**, silencing NELL2 could significantly increase GC cells migration and invasion. While migration and invasion of GC cells were impaired by NELL2 overexpressing. The transfection efficiency of overexpressing NELL2 and knockdown NELL2 was shown in **Figure S1F**.

Then qRT-PCR was performed to examine the expression of NELL2 relative to tRF-3017A. We found that mRNA level of NELL2 was downregulated in cancer tissues compared with NATs among 84 cases (three cases did not participate in this analysis due to insufficient cDNAs) of GC tissues (**Figure 4G**). Correlation between expression level of tRF-3017A and NELL2 was analyzed and the correlation coefficient was -0.41 (P < 0.001) (**Figure 4H**), which suggesting a moderate negative relevance between tRF-3017A and NELL2 RNA expression levels.

In order to verify whether tRF-3017A regulates NELL2 through miRNA-like mRNA silencing mechanism, luciferase report assay and RIP assay were performed. Luciferase reporter assay based on complementary pairing in the 3 ‘UTR region of NELL2 (**Figure S1G**) showed that relative luciferase activity of luciferase mRNA containing the wild-type NELL2-3’UTR could significantly increased or decreased by tRF-3017A inhibitor or mimics. In contrast, mutant NELL2-3’UTR luciferase activity was not affected (**Figure 4I**). These results demonstrated that tRF-3017A could specifically bind 3’UTR of NELL2. Western Blot experiments for 12 patients with significant differential tRF-3017A expression from the study cohort showed that the expression of tRF-3017A inversely correlated with NELL2 (**Figure S1H**). Furthermore, RIP based on Ago2 was performed in GC cells to explore the level of tRF-3017A and NELL2 immunoprecipitated by Ago2 *via* qRT-PCR. The results of Ago2-RIP assay validated that both tRF-3017A and NELL2 interact directly with Ago2 (**Figure 4J**).

### The Migration and Invasion Ability of GC Cells Which Effected by tRF-3017A Could Be Largely Rescued by Inhibition or Overexpression of NELL2

**Figure 5:**
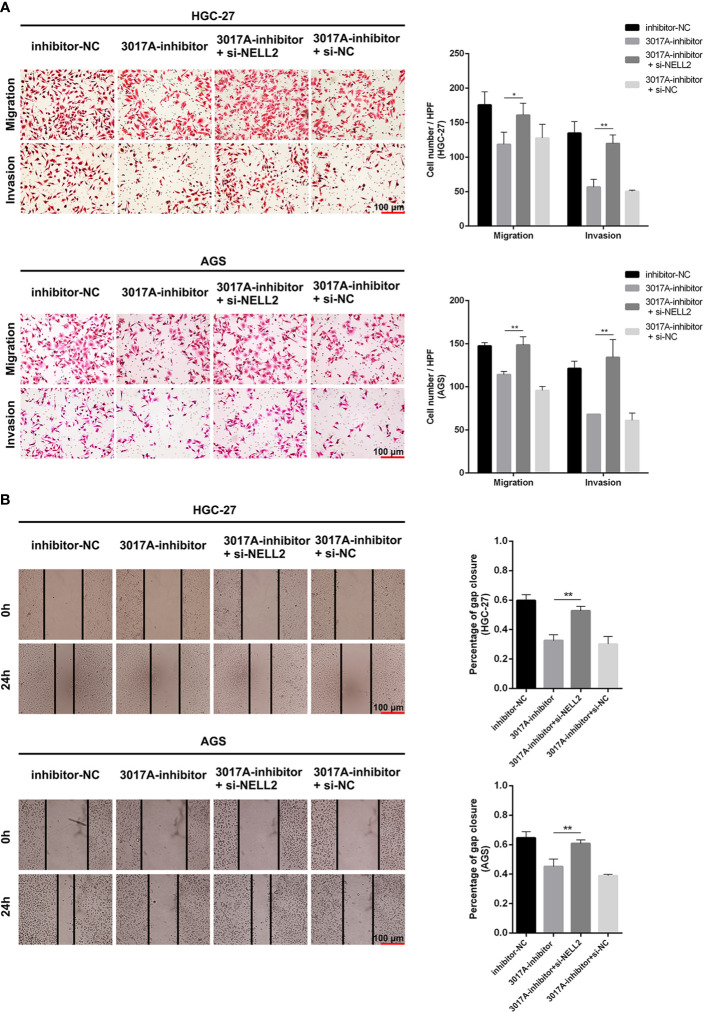
Knockdown of NELL2 restores impaired migration and invasion ability induced by knocking down tRF-3017A. **(A)** GC cell migration and invasion ability were detected after co-transfected with tRF-3017A inhibitor and si-NELL2. **(B)** The scratch wound healing assay was used to detect GC cell migration ability after co-transfection. Data are shown as mean ± SD. NC, negative control. *P < 0.05; **P < 0.01.

**Figure 6:**
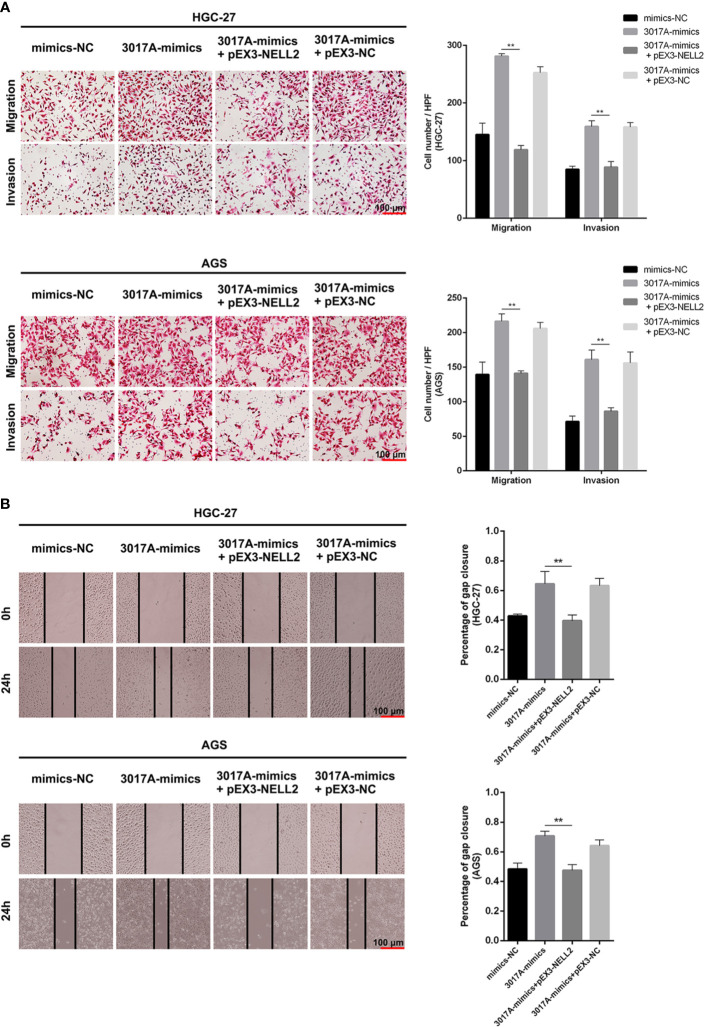
Enhanced ability of migration and invasion induced by overexpression of tRF-3017A was restored by overexpressing NELL2. **(A)** GC cell migration and invasion ability were detected after co-transfected with tRF-3017A mimics and pEX3-NELL2. **(B)** The scratch wound healing assay was used to detect GC cell migration ability after co-transfection. Data are shown as mean ± SD. NC, negative control. **P < 0.01.

**Figure 7:**
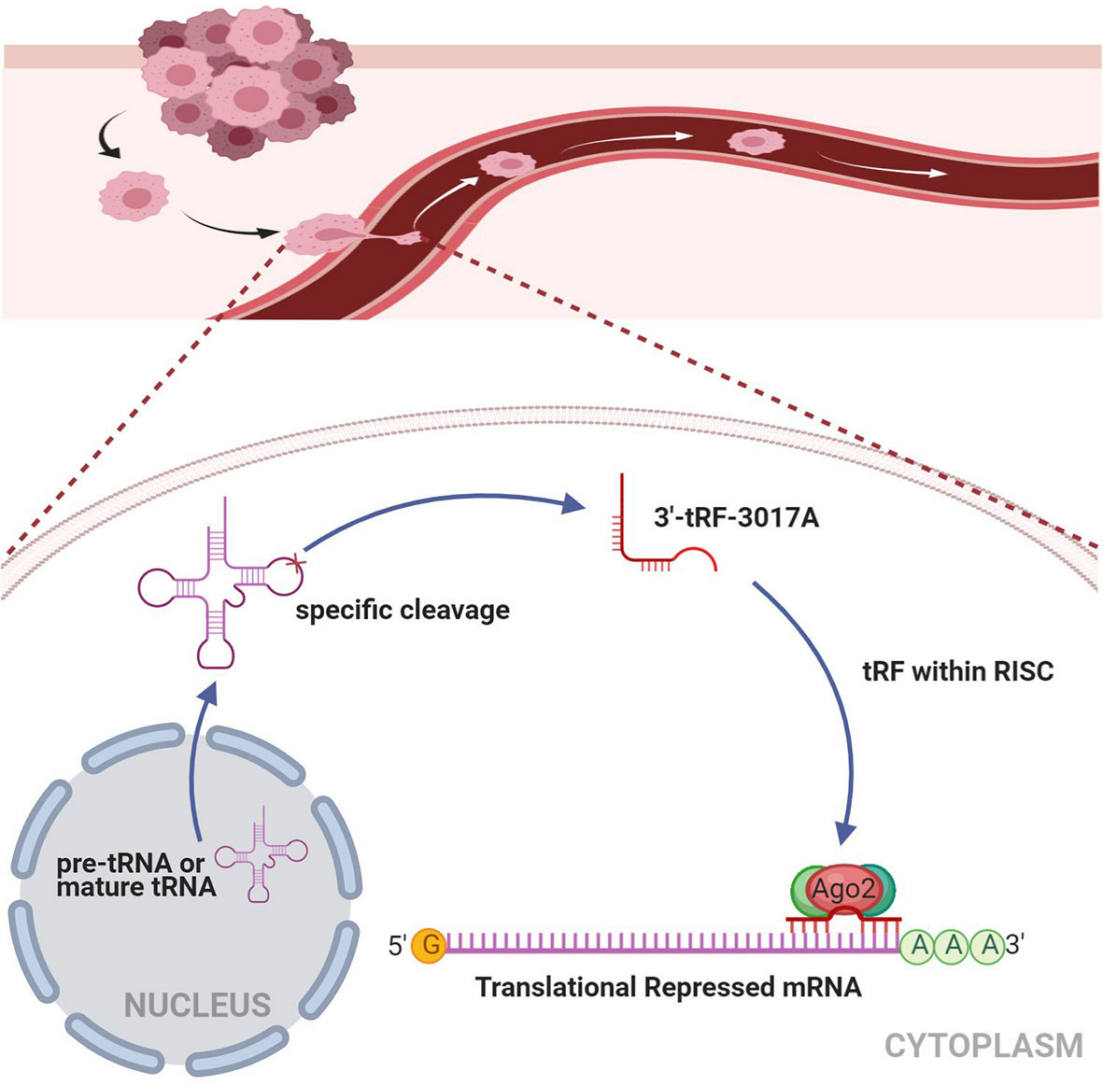
Working model illustrating the mechanism in which tRF-3017A may promote GC cell migration and invasion by targeting NELL2. RISC, RNA-induced silencing complex.

Rescue experiments were performed to further support the results that tRF-3017A affects the ability of migration and invasion of GC cells by targeting NELL2. Our results showed that knockdown of NELL2 restores impaired migration and invasion ability induced by knocking down tRF-3017A (**Figures 5A, B**). Likewise, the enhanced ability of migration and invasion induced by overexpression of tRF-3017A was reversed by overexpressing NELL2 (**Figures 6A, B**). In conclusion, our results demonstrated that tRF-3017A may be in action in promoting GC cells migration and invasion by regulating its downstream target gene NELL2. This presents a potential mechanism by which tRF-3017A promotes the invasion and migration of GC cells (**Figure 7**).

## Discussion

The development of GC is accompanied by the gradual accumulation of multiple genes and epigenetic changes in a complex regulatory interaction network (47, 48). Early studies suggested that tRFs are the products of random degradation of tRNA (49, 50). With the advance of sequencing technology, we gradually realized that tRFs play crucial roles in different sorts of biological processes and that they may play an important role in tumorigenesis and progression. Therefore, the biological functions of these tRFs have attracted growing attention. Increasing evidence shows that tRFs play important roles in oncogenesis and the development of various tumors (51–53). tRF-1001 is essential for proliferation in colorectal cancer, and knocking down tRF-1001 arrests tumor cells in the G2 phase (14). Stable expression of tRF-CU1276 can inhibit a DNA dynamics regulator, endogenous RPA1, thus regulating the molecular DNA damage response and inhibiting proliferation in lymphoma cell lines (54). The newly identified tRF-1280, previously known as miR-1280, can inhibit metastasis in colorectal cancer (36). Moreover, the metastasis and progression of breast cancer can be inhibited by endogenous tRFs replacing YBX1 (23). Nevertheless, the expression level, potential function, and molecular mechanism of tRFs in GC are still indistinct.

In the present study, in order to find the differentially expressed tRNA-derived fragments in GC, we performed tRF&tiRNA microarray analysis on 10 pairs of GC and its paired NATs. We selected tRF-3017A which is upregulated for further study. We detected tRF-3017A expression level and found that it was abnormally highly expressed in GC tissues and cell lines. To identify the relevance between tRF-3017A and clinicopathologic features, the chi-square test was performed and suggested the association between higher expression of tRF-3017A and lymph node metastasis. It is well known that the occurrence of GC metastasis is a key factor affecting patient prognosis. Therefore, we explored the diagnostic value of tRF-3017A for occurrence and lymph node metastasis of GC through ROC analysis. Unfortunately, tRF-3017A has not demonstrated satisfactory diagnostic efficacy. The aberrant tRF-3017A expression level in GC tissues and cell lines has never been reported. Thus, in this study, we are the first to reveal the association between tRF-3017A and GC lymph node metastasis.

In terms of mechanisms, previous studies have shown that tRFs play important roles in RNA silencing through the complement of tRFs and target mRNA. 3’ tRFs were found to bind with Ago2, which is important in RNA interference (RNAi), for target recognition (26, 55, 56). Some studies indicate that tRFs bind directly to target mRNA, resulting in microRNA like effects (57, 58). For example, tRF-CU1276 in B-cell lymphoma regulates the molecular response to DNA damage by directly binding to the 3’UTR of RPA1 (54). tRF5-Glu was found to inhibit cell proliferation in ovarian cancer through directly binding to the 3’ UTR of BCAR3 (59). In this present study, it was observed through functional experiments that the migration and invasion ability of GC cells were promoted by the increase of tRF-3017A and also inhibited by the absence of tRF-3017A. Based on the reported RNAi mechanism, we further explored the mechanism of tRF-3017A inducing GC metastasis. According to the 3’ UTR complementary combination principle, the mRNA target prediction algorithm, the reference of existing research and validation of RNA and protein levels, we selected the possible regulated target genes, NELL2, downstream of tRF-3017A. Existing studies have shown that NELL2 is enriched in paracancer tissue and can inhibit clear cell carcinoma metastasis. Therefore, we attempted to verify whether NELL2 participated in the mechanism promoting GC metastasis of tRF-3017A. Correlation analysis showed that there is a moderate negative correlation between tRF-3017A and NELL2 expression. Knockdown and overexpression experiments validated that NELL2 could inhibit the migration and invasion of GC cell. Further luciferase report analysis and RIP-Ago2 assay demonstrated that tRF-3017A could form complex with Ago2 to target silencing NELL2. In addition, results of RESCUE experiments supported that tRF-3017A affects the migration and invasion of GC cells by regulating its downstream target gene NELL2. Our results show that tRF-3017A specifically bind to the 3’ UTR of NELL2 by interacting with Ago2 and negatively regulate NELL2 expression, similar to the mechanism of miRNA-mediated target gene silencing. In summary, this study verified that a tRNA derived fragment, tRF-3017A, can induce GC metastasis by targeting NELL2. The mechanism of tRF-3017A induced metastasis in which NELL2 is involved may provide a new therapeutic target for inhibiting GC metastasis.

## Conclusions

In conclusion, this study found that tRF-3017A is abnormally highly expressed, revealed that the upregulated expression of tRF-3017A is associated with lymph node metastasis in GC and demonstrated that tRF-3017A promotes migration and invasion of GC cell lines. Moreover, we revealed that tRF-3017A promotes GC through regulation of NELL2 in a mechanism similar to miRNA-mediated target gene silencing.

## Data Availability Statement

The original contributions presented in the study are included in the article/**Supplementary Material**. Further inquiries can be directed to the corresponding authors.

## Ethics Statement

The studies involving human participants were reviewed and approved by Animal Care and Use Committee of the First Affiliated Hospital of China Medical University. The patients/participants provided their written informed consent to participate in this study.

## Author Contributions

LHT wrote the main manuscript and performed the experiments. LHT, YXS, and ZNW designed the research. LHT, XWC, and JXS performed data analysis. ZHW, FZ, and JXS contributed to manuscript revisions. All authors contributed to the article and approved the submitted version.

## Funding

This work was supported by the project of Natural Science Foundation of China (81872031), Major R&D Plan of Liaoning Province (2019JH1/10300007), Xingliao Talents Program in Liaoning Province (XLYC1807164), Scientific and technological innovation talents program of Shenyang (RC190202).

## Conflict of Interest

The authors declare that the research was conducted in the absence of any commercial or financial relationships that could be construed as a potential conflict of interest.

## Supplementary Material

Supplementary Figure 1
**(A)** Heatmap for statistically significant gene results in 10 paired GC tissues and matched NATs. **(B)** ROC curve of all GC patients based on tRF-3017A expression in tumor tissue samples and matched NATs. **(C)** ROC curve of GC patients with/without lymph node metastasis. **(D)** Transfection efficacy was detected after transfection with tRF-3017A mimics in HGC-27 and AGS cells. **(E)** GC cell proliferation was detected after transfection with tRF-3017A mimics or inhibitor by CCK-8 assay. **(F)** NELL2 mRNA and protein levels in GC cells transfected with si-NELL2 oligo or pEX3-NELL2 plasmid. **(G)** 2D structure diagram based on base complementary pairing principle. Data are shown as mean ± SD. NC, negative control. *P < 0.05; **P < 0.01. **(H)** Analysis of NELL2 protein in 12 GC tissues and their NATs by Western blot. GAPDH protein was used as an endogenous reference. The intensity of each band was densitometrically quantified. Values under each paired tissue sample indicated the fold change of level of NELL2 protein in cancer tissues relative to NATs. T, tumor tissue; N, NAT; T/N (tRF-3017A), the expression of tRF-3017A in cancer tissues relative to NATs.

Supplementary Data Sheet 1 Raw Data for qRT-PCR.

Supplementary Data Sheet 2 Differentially expressed genes in microarray analysis.
