# Supplementary figures and images for: The tRNA-Derived Fragment-3017A Promotes Metastasis by Inhibiting NELL2 in Human Gastric Cancer
Source: Front Oncol. 2021 Feb 16;10:570916. doi: 10.3389/fonc.2020.570916 (PMC7921707; doi:10.3389/fonc.2020.570916)

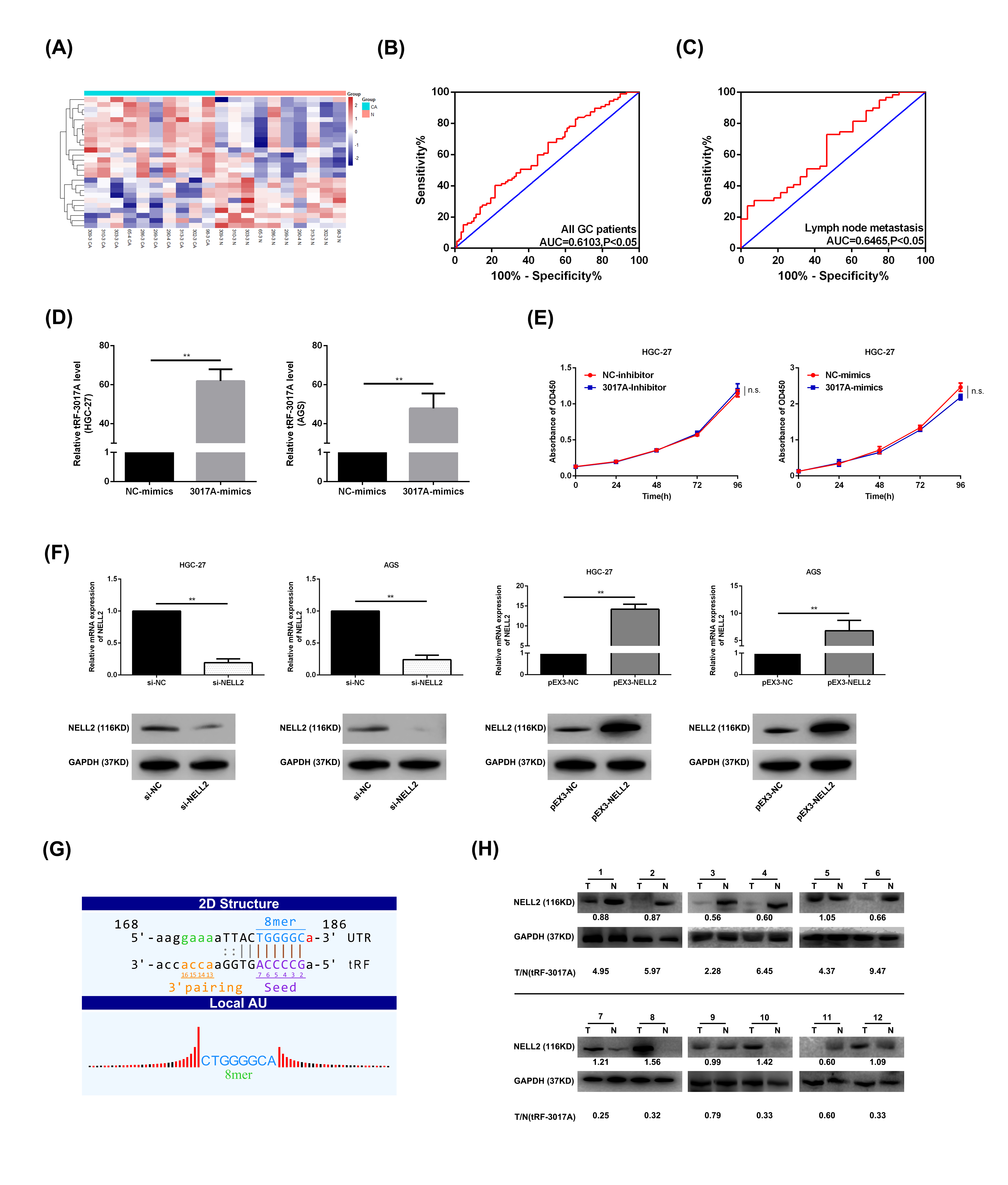

Supplement: Supplementary Figure 1 — (A) Heatmap for statistically significant gene results in 10 paired GC tissues and matched NATs. (B) ROC curve of all GC patients based on tRF-3017A expression in tumor tissue samples and matched NATs. (C) ROC curve of GC patients with/without lymph node metastasis. (D) Transfection efficacy was detected after transfection with tRF-3017A mimics in HGC-27 and AGS cells. (E) GC cell proliferation was detected after transfection with tRF-3017A mimics or inhibitor by CCK-8 assay. (F) NELL2 mRNA and protein levels in GC cells transfected with si-NELL2 oligo or pEX3-NELL2 plasmid. (G) 2D structure diagram based on base complementary pairing principle. Data are shown as mean ± SD. NC, negative control. *P < 0.05; **P < 0.01. (H) Analysis of NELL2 protein in 12 GC tissues and their NATs by Western blot. GAPDH protein was used as an endogenous reference. The intensity of each band was densitometrically quantified. Values under each paired tissue sample indicated the fold change of level of NELL2 protein in cancer tissues relative to NATs. T, tumor tissue; N, NAT; T/N (tRF-3017A), the expression of tRF-3017A in cancer tissues relative to NATs. [file Image_1.jpeg]
