# Supplementary material for: The tRNA-Derived Fragment-3017A Promotes Metastasis by Inhibiting NELL2 in Human Gastric Cancer
Source: Front Oncol. 2021 Feb 16;10:570916. doi: 10.3389/fonc.2020.570916 (PMC7921707; doi:10.3389/fonc.2020.570916)
Supplement: Supplementary file 4 [file Table_1.docx]

**Table S1 List of sequence in this study.**

| Name | Sequence (5’ - 3’) |
| --- | --- |
| tRF-3017A Forward | AGCCCCAGTGGAACCACC |
| U6 Forward | GGAACGATACAGAGAAGATTAGC |
| U6 Reverse | TGGAACGCTTCACGAATTTGCG |
| NELL2 Forward | GCCTTGCCCAGATGTGGAGTG |
| NELL2 Reverse | TGTCATTGCGGATGGTGTCAGC |
| GAPDH Forward | TGAACGGGAAGCTCACTGG |
| GAPDH Reverse | TCCACCACCCTGTTGCTGTA |
| tRF-3017A mimics | Sense：  AGCCCCAGUGGAACCACCA  antisense：  GUGGUUCCACUGGGGCUUU |
| NC-mimics | Sense：UUCUCCGAACGUGUCACGUTT  antisense：ACGUGACACGUUCGGAGAATT |
| tRF-3017A inhibitor | UGGUGGUUCCACUGGGGCU |
| NC-inhibitor | CAGUACUUUUGUGUAGUACAA |
| NELL2 siRNA | GAGCCTGTATTGCCGCTAA |

**Abbreviations:** NC, negative control.
